# Investigating the role of predictive death anxiety in the job satisfaction of pre-hospital emergency personnel during the COVID-19 pandemic

**DOI:** 10.1186/s12873-022-00762-x

**Published:** 2022-12-06

**Authors:** Najme Chegini, Soheil Soltani, Sajad Noorian, Mostafa Amiri, Fatemeh Rashvand, Saeed Rahmani, Mohadese Aliakbari, Mojtaba Senmar

**Affiliations:** 1 Student Research Committee, Qazvin University Of Medical Science, Qazvin, Iran; 2 Emergency Medical Service Center, Qazvin University of Medical Sciences, Qazvin, Iran; 3 Department of Statistics, Faculty of Science, University of Qom, Qom, Iran; 4 Student Research Committee, Social Determinants of Health Research Center, Research Institute for Prevention of Non–Communicable Diseases, Qazvin University of Medical Sciences, Qazvin, Iran

**Keywords:** Job satisfaction, Emergency medical service, Anxiety, COVID-19, Death

## Abstract

**Background:**

Pre-hospital emergency staffs as the frontline forces fighting against COVID -19 have been affected by this pandemic. Today, the occupational and mental health of these individuals is particularly important to the health care system. Death anxiety is one of the inevitable things in this job, and not paying attention to it can cause unwanted effects such as changing the level of job satisfaction of the personnel. The purpose of this study was to determine the role of predictive death anxiety in the job satisfaction of pre-hospital emergency personnel during the COVID-19 pandemic.

**Methods:**

This cross-sectional descriptive study was conducted among pre-hospital emergency staffs in Qazvin Province, Iran in 2021–2022. Among the bases chosen by the census method, 198 samples were included in the study by the available method. Data collection tools included the Demographic Checklist, Templer's Death Anxiety scale, and the Minnesota Job Satisfaction Questionnaire. The data were analyzed with descriptive and inferential statistics and SPSS 20 software.

**Results:**

The mean age of the samples was (33.14 ± 6.77). 167 persons were male and the others were female. The average job satisfaction and death anxiety of the personnel were 55.07 ± 11.50 and 8.18 ± 1.96, respectively. Pearson's correlation coefficient between the two variables was r = -0.126 And a null correlation coefficient hypothesis has been confirmed with *p*-value = 0.077.

**Conclusions:**

The results showed a high level of death anxiety and average job satisfaction. Although these two variables do not have a significant relationship with each other, considering that they do not have the appropriate level, it needs more investigation and consideration.

**Supplementary Information:**

The online version contains supplementary material available at 10.1186/s12873-022-00762-x.

## Background

The COVID-19 pandemic remains a global threat and has impacted over 200 countries and regions both economically and humanely [1]. The effects of this disease vary from asymptomatic infection to severe pneumonia and respiratory failure [2]. Health care workers are the front-line fighters in the treatment of COVID-19 [3]. Otherwise, the personnel of the pre-hospital emergency services system were among the first groups to be mobilized to deal with, transfer, and treat patients with COVID-19 [4]. Because this system plays a vital role in providing timely care to patients [5]. In this system, there are trained and authorized persons working in an aerial or ground emergency [6]. During their workday, these staffs faces a wide range of mental injuries and harms in their patients [7]. In the current situation of the COVID-19 pandemic, the increasing number of confirmed and suspected cases of coronavirus and the high work pressure have led to psychological pressure on pre-hospital emergency personnel [8]. In other words, they are exposed to various mental disorders due to stressful factors such as increased workload, increased number of patients, frequent exposure to death of patients, and fear of getting infected and transmitting the disease to their friends [9].

Death anxiety is one of those conditions [9]. Death anxiety is a multi-dimensional concept with emotional, cognitive, and experiential features and is defined as a sense of anxiety or fear of death [9]. It has been stated that providing care to a dying person is one of the most stressful human experiences [10], so it can be said that facing death and death anxiety is inevitable in the profession of emergency medicine [11]. Although the behavior of pre-hospital emergency staffs in relation to death anxiety is not yet fully known [10], But it must be said that the death anxiety can be an important threaten for job satisfaction of emergency staff [12].

Job satisfaction is fundamentally linked to human needs and is the result of an individual's different attitudes of a person toward work [13]. Job satisfaction is a challenge for health care organizations that can affect patient safety, performance and usefulness, employee morale, early retirement, commitment to the organization, and above all, patient satisfaction [14]. There is a consensus among several models that explain job satisfaction, and it is that job satisfaction is influenced by external factors such as working conditions and internal factors such as self-efficacy beliefs [15]. Therefore, it should be said that good management of psychosocial risks is related to the elements of job satisfaction and acts as a protective factor against the risks of work stress [16]. Therefore, job satisfaction and its related factors are very important to emergency staffs and have a significant impact on their work [17].

In general, pre-hospital emergency personnel has witnessed the suffering and death of patients through the COVID-19 pandemic [18]. Despite the importance of this person in the management of this pandemic, limited research has been done in this field. Apart from this infection of COVID-19 in this staffs and the unavoidable impact of this phenomenon on the health of human resources, the variables of death anxiety and job satisfaction are more important in pre-hospital emergency personnel. Therefore, the present study was conducted to determine the role of predicting death anxiety in the job satisfaction of emergency medical staffs in Qazvin Province during the COVID-19 Pandemic.

## Methods

### Setting and study design

This study was a cross-sectional and analytical descriptive that was conducted in January 2021–2022 August among pre-hospital emergency personnel in Qazvin Province of Iran. All the bases of the province (48 bases) were included in the census study and then the workers were included in the study using the available method. The sample size was determined using Cochran's formula of 168 persons, and with a 10% drop-out potential, the minimum sample size was 185 persons. The inclusion criteria were not having a confirmed psychological illness and not being willing to join the study. If the questionnaires were not completely completed, the sample was excluded from the study.

### Data collection tools

The Demographic checklist, Templer's 15-question death anxiety scale, and Minnesota's 19-question job satisfaction questionnaire were used to collect data. The demographic checklist included information about age, gender, education level, marriage, history of first-degree family members being infected with COVID-19, having an underlying disease, economic status, the person's role in the family, and the person's history of being infected with COVID-19. Information about job satisfaction was collected through the Minnesota Job Satisfaction Questionnaire. This questionnaire consists of 19 items and 6 subscales of the Payment System (3 questions), Job Type (4 questions), Advancement Opportunities (3 questions), Organizational Atmosphere (2 questions), Leadership Style (4 questions), and Physical Conditions (3 questions). The scoring of the Minnesota Job Satisfaction Questionnaire is in the form of a Likert scale, with 1, 2, 3, 4, and 5 points for the options "I completely disagree", "I disagree", "I have no opinion", "I agree" and "I completely agree". Scores between 19 and 38 indicate a low level of job satisfaction. Scores between 38 and 57 indicate an average level of job satisfaction. Scores above 57 indicate a very good level of job satisfaction. The reliability of the questionnaire was obtained by calculating Cronbach's alpha coefficient of 0.75 and the validity and reliability of the questionnaire were generally considered to be favorable [19].

Templer's Death Anxiety scale was used to investigate death anxiety. This tool includes 15 two-choice questions (yes or no) in 5 dimensions Fear of Death [1, 12, 14], Fear of Pain and Disease [2, 4, 6, 13], Thoughts Related to Death [5, 9, 19], Passing Time and Short Life [3, 7, 10] and Fear of the Future [8, 15], which measures the subject's attitudes towards death. An affirmative answer is a sign of anxiety in an individual. The range of scores of the questionnaire is from zero to fifteen, and higher scores (higher than the average score of "8 points") indicate a high level of death anxiety in staffs. The scoring method is such that 1 point is given for each correct option and 0 points for each wrong option. The validity and reliability of the questionnaire have been confirmed [20].

### Analyze

The data collected were analyzed by the SPSS version 22 and using descriptive indicators such as mean, standard deviation, number, and percentage. Pearson's correlation coefficient was used to examine the relation between job satisfaction and the predictive level of death anxiety. To examine the difference in the mean of the dependent variables between the defined groups, an independent t-test was used for two-mode independent variables, and a one-way analysis of variance was used for independent variables with more than two modes. The significance level for all tests was considered to be 0.05 or lower.

## Results

One hundred ninety-eight Pre-hospital emergency staffs with an average age of 33.14 ± 6.77 were included in the study. Among them, 167 were male and the others were female. The study samples included 147 single persons and the others were married ones. Of the samples, 10 staffs had a master's degree, 93 had a bachelor's degree, 83 had an associate's degree, and the remainder had a diploma. In the study samples, 25 staffs had back and neck disc disease, 5 staffs had hypertension, 3 ones had heart disease, one person had kidney disease, and one person had diabetes. The remaining samples showed no underlying illness. Table 1 shows other demographic characteristics.

**Table 1:** The demographic characteristics of participants

Demographics characteristics	Number	percent
History of first-degree family members being infected with COVID-19		
YES	148	74.7
NO	50	25.3
Person's history of being infected with COVID-19		
YES	130	65.7
NO	68	34.3
Economic status		
POOR	95	48
MEDIUM	96	48.5
GOOD	7	3.5
The person's role in the family ( head of the family)		
YES	132	66.7
NO	66	33.3

The results of this study showed that the mean job satisfaction and death anxiety of the personnel were 55.07 ± 11.50 and 8.18 ± 1.96, respectively. In other words, the job satisfaction of the staffs is average and their death anxiety is high. Table 2 shows the mean scores on each sub-scale for job satisfaction and death anxiety. The results of the study in response to determining the predictive role of death anxiety in job satisfaction showed that the Pearson correlation coefficient between the two variables is *r* = -0.126 (Additional file 1). This negative value indicates that as the level of anxiety increases, the level of job satisfaction decreases on average. But this value for the correlation coefficient shows a low linear relation between the two variables. The assumption of a null correlation factor is confirmed with *p*-value = 0.077 (Additional file 2). Therefore, the correlation is not significant and the variance analysis for the regression also supports this case.

**Table 2:** Mean and standard deviation of death anxiety scores and job satisfaction of pre-hospital emergency personnel

Subscales Scale	Minimum	Maximum	Mean Std. Deviation
Payment System	3.00	13.00	6.22 ± 2.39
Job Type	6.00	20.00	13.68 ± 3.07
Advancement Opportunities	3.00	15.00	7.78 ± 3.11
Organizational Atmosphere	2.00	10.00	6.30 ± 1.86
Leadership Style	4.00	18.00	11.67 ± 2.65
Physical Conditions	3.00	15.00	9.31 ± 2.86
Total Job Satisfaction	23.00	86.00	55.07 ± 11.50
Fear of Death	.00	3.00	1.47 ± .63
Fear of Pain and Disease	.00	4.00	2.51 ± .88
Thoughts Related to Death	.00	3.00	1.57 ± .65
Passing Time and Short Life	.00	3.00	1.76 ± .82
Fear of the Future	.00	2.00	.84 ± .53
Total Death Anxiety	4.00	13.00	8.18 ± 1.96

The results of the independent t-test showed that there is no significant relation between death anxiety and job satisfaction with gender, age, marital status, and history of COVID-19 in first-degree family members (Additional file 3A1-4). While average job satisfaction and death anxiety scores are higher for females than males and singles than others. The average scores of job satisfaction and death anxiety in the group of personnel without a history of COVID-19 in first-degree family members are higher than in others, and there is a non-significant and weak positive relationship between the age variable and these two indicators.

The average scores of job satisfaction in staffs without underlying disease are higher than those with underlying disease, and the results of the independent t-test also show that this difference is significant at the 0.05 level (*p*-value = 0.01). The average scores of death anxiety in staffs without underlying disease are lower than in those with underlying disease. However, this difference was not significant (Additional file 4).

The results showed that the average job satisfaction scores of staffs who had a history of being infected with COVID-19 are lower than those without a history of being infected. The results of the independent t-test show that this difference is significant at the 0.05 level (*p*-value = 0.01). The average scores of death anxiety in the group of staffs with a history of COVID-19 are higher than those without a history. But the independent t-test results show that this difference is not significant at the 0.05 level (*p*-value = 0.74) (Additional file 5).

In this study, staffs with a Diploma have the highest average job satisfaction score and other staffs with a master's degree have the lowest average job satisfaction score. In the case of death anxiety, it is the opposite. But the unidirectional ANOVA test showed that these differences are not significant (Additional file 6). The results showed that persons with an average economic status have the highest average job satisfaction and death anxiety score. The results of the one-way analysis of variance show that the average difference in job satisfaction scores in three groups with poor, average, and good economic status is significant at the 0.05 level (*p*-value < 0.000), but the average difference in death anxiety scores in three groups is not significant at the 0.05 level (*p*-value = 0.744) (Additional file 7).

In addition, the results showed that the average scores of job satisfaction and death anxiety in the group of staffs who are not the head of the household are higher than those who are the head of the household. The independent t-test results show that this difference is not significant at the 0.05 level (Additional file 8).

## Discussion

The findings of the current research, which investigated the role of predictive death anxiety in the job satisfaction of pre-hospital emergency personnel during the spread of the COVID-19 disease, showed that the level of job satisfaction of the staffs is average and their death anxiety is high. Although death anxiety and job satisfaction in the study samples during the COVID-19 Pandemic have a low inverse linear relation but have no significant correlation.

In the study of Kojaei et al. in Iran [9], the average death anxiety score of emergency personnel was 5.68, which is lower compared to the average death anxiety in the present study. In other words, the results differed from this study. However, both studies were done during the COVID-19 Pandemic. Pre-hospital emergency staffs has faced various challenges with the outbreak of COVID-19 and they experience significant stress because they are the first staffs to attend the bedside of COVID-19 patients [21]. It seems that the pre-hospital emergency personnel of Qazvin is more impacted by the COVID -19 pandemic than the personnel mentioned above. According to this study, the level of death anxiety of pre-hospital emergency personnel was higher than average in the study by Esadi et al. [22]. It appears that the completion of both studies during the COVID-19 Pandemic is a common reason for the high level of death anxiety in the samples from both studies. In the meta-analysis study of Sahebi et al. [23], which investigated post-traumatic stress disorder (PTSD) among health service workers during the COVID-19 Pandemic, the prevalence of PTSD among these workers was reported to be high (13.52%). Although the variables of the above study are different from the variables of the present study, considering that PTSD and death anxiety has somewhat similar concepts, it can be said that the results of the above study are somewhat consistent with the results of the present study. In addition, Adibi et al.'s study [24] also reported a relatively high prevalence (30.5%) of anxiety disorders among health service workers during the COVID-19 Pandemic. Although the above study was conducted by meta-analysis method, in the present study, the level of death anxiety of the personnel was high, which shows that the results of the two studies are in line with each other.

Kasraei et al.'s study before the outbreak of COVID-19 Pandemic showed that job satisfaction has no significant relation to death anxiety [25], which is in line with the findings of the present study. Although the relation was not significant, there was an inverse correlation between the two variables, and that opposite relation seems logical. According to Aydin et al. [26], it has been found that death anxiety negatively affects life satisfaction. Although the above study did not consider job satisfaction, in the sense that death anxiety has a relation to job satisfaction, it is somewhat consistent with the results of the present study.

The results of the study by Kamil et al. [27] showed that there is an inverse and low-significance relationship between death anxiety and life satisfaction. Although unlike the present study, the relationship between the two variables is significant, in line with the results of the present study, it shows that death anxiety and the concept of satisfaction have an inverse relationship. The results of Özmen et al.'s study [28] showed that there is an inverse and weak relationship between the fear of Covid-19 and life satisfaction. Although the population of the above study was not healthcare personnel and did not consider job satisfaction, in principle, the inverse relationship between death anxiety and the life satisfaction variable is somewhat in line with the results of the present study. In general, it should be said that most studies have addressed the factors that cause death anxiety, but the consequences of death anxiety such as job satisfaction have not been attentioned.

In the research by Kajaei et al. [9], the level of death anxiety was found to be higher among females than males, consistent with the results of this study. Differences in mental health among males and females can be attributed to gender differences that are embedded in genetics, anatomy, and physiology [29]. As a result, given these differences between males and females, the increase in death anxiety scores among females may be justified. In Sadeghi et al.'s study [11], it was found that age, marital status, and educational level have a significant relation to death anxiety, which is not consistent with the results of the present study. Furthermore, in this study, death anxiety scores were higher among those who are not heads of families. This result is partially consistent with the high death anxiety scores among single persons. Because the study by Sadeghi et al. was done before the COVID-19 outbreak, it can explain this difference to some extent. It seems that considering the key role of emergency medical staffs in the COVID-19 pandemic, various stressful factors have imposed many psychological effects on this personnel [9] and led to the creation of such differences before and after the outbreak. In the current study, death anxiety was higher in the group of staffs without a history of first-degree family members being infected with the COVID-19 disease than staffs with a history of first-degree family members being infected with the COVID-19 disease. Health care workers are concerned about family members, many of whom have separated from their families to prevent the spread of the disease [30]. It appears that the reason for the high rates of death anxiety in this group of individuals is because of the fear of disease transmission. In addition, those who have contracted COVID-19 themselves had more death anxiety scores than those who did not. The continued spread of the disease, high morbidity, and mortality cause fear and anxiety and negatively impact staffs 's mental health. This applies to COVID-19 as well. Because assessing the psychological effects of the COVID-19 outbreak in terms of psycho-social health shows that staffs experience the highest level of stress or anxiety [31]. Life-threatening illnesses are associated with some existential problems that have psychological implications such as intrusive thoughts about death [32]. For this reason, it can be explained why the death anxiety scores are lower in samples with no underlying disease history than in others.

In agreement with this study, in the study by Basaber et al. [33], it was also found that there was a moderate level of job satisfaction among emergency medical staffs. However, in the above study, job satisfaction is closely related to gender and it is not in line with the findings of this study. In the study by Sonems et al. [34], the overall level of job satisfaction of emergency physicians was rated as high, which is inconsistent with the results of this study. It should be noted that pre-hospital emergency has led to the creation of special conditions for this staffs due to special environmental conditions, time constraints, road traffic, the openness of the workspace, and the expectations of the companions [33] that it can be said that job satisfaction impacted by these factors, so we see the difference in the results of the two studies. Although the samples of this study are different from those in the Sonmez et al. study. In Yu et al. research [35], job satisfaction was found to have no significant relation to age and marital status, which is consistent with the results of this study. Although in Yu et al.'s study, job satisfaction increases with the increase in the education level of staffs, and this relation is significant, in the present study, staffs with a master's degree have a low level of job satisfaction. It seems that in the Qazvin pre-hospital emergency personnel community, the existing facilities have not been able to meet the expectations of staffs with higher qualifications, and for this reason, these staffs express low job satisfaction. In the study of Elatif et al. among Egyptian doctors [36], it was found that the fear of COVID-19 Pandemic has a negative relation with job satisfaction, in other words, it can be said that the results of the above study are somewhat consistent with the present study. Because in this study, staffs without a history of COVID-19 and those with first-degree family members who were not infected with the coronavirus were more satisfied with their job than others. In the study by Jaradi et al. low job satisfaction was found to be associated with low back pain [37]. In the present study, those without underlying illness expressed a higher level of job satisfaction. In other words, there is consistency between the two studies.

### Limitations

One of the limitations of this research is the generalization of the results because the structure of pre-hospital emergencies based on a diversity of missions differs not only from one country to another but also in the cities of the same province. However, in this study, we attempted to resolve this limitation by sampling from every base in the province.

### Strengths

Sampling from all bases of the province in the dispersion and different nature of the missions was one of the strengths of the present study.

## Conclusion

The findings of this study provided valuable information about the level of death anxiety and job satisfaction of pre-hospital emergency staffs during the COVID-19 pandemic. Considering the high level of death anxiety of the staffs, it is suggested to implement psychological interventions and training to reduce the negative effects of the COVID -19 pandemic in this very diverse environment. Decision makers and related managers are expected to improve staffs job satisfaction by providing opportunities for advancement, an organizational atmosphere, physical conditions, and an appropriate leadership style. It is suggested that in future studies, a comparative study of variables between pre-hospital emergency personnel and hospital emergency staffs should be conducted. The results of this study may be used in the environment and context similar to the pre-hospital emergency of Qazvin.

## Supplementary Information

**Additional file 1. **Pearson correlation coefficient between death anxiety and job satisfaction.

**Additional file 2.** Anova for Regression.

**Additional file 3. **A1. Independent Samples Test. A2. Pearson's R. A3. Independent Samples Test. A4. Independent Samples Test.

**Additional file 4.** Independent Samples Test.

**Additional file 5.** Independent Samples Test.

**Additional file 6.** ANOVA.

**Additional file 7.** ANOVA.

**Additional file 8.** Independent Samples Test.

## Data Availability

Data are available by contacting the corresponding author.

## Abbreviation

PTSD: Post-traumatic stress disorder
