# Supplementary material for: Investigating the role of predictive death anxiety in the job satisfaction of pre-hospital emergency personnel during the COVID-19 pandemic
Source: BMC Emerg Med. 2022 Dec 6;22:196. doi: 10.1186/s12873-022-00762-x (PMC9727867; doi:10.1186/s12873-022-00762-x)
Supplement: Supplementary file 1 — Additional file 1. Pearson correlation coefficient between death anxiety and job satisfaction. [file 12873_2022_762_MOESM1_ESM.docx]

| Additional file 1. Pearson correlation coefficient between death anxiety and job satisfaction | | | | | |
| --- | --- | --- | --- | --- | --- |
|  | | Value | Asymptotic Standard Error^a^ | Approximate T^b^ | Approximate Significance |
| Interval by Interval | Pearson's R | -.126 | .075 | -1.781 | .077^c^ |
| N of Valid Cases | | 197 |  |  |  |
