# Supplementary material for: Investigating the role of predictive death anxiety in the job satisfaction of pre-hospital emergency personnel during the COVID-19 pandemic
Source: BMC Emerg Med. 2022 Dec 6;22:196. doi: 10.1186/s12873-022-00762-x (PMC9727867; doi:10.1186/s12873-022-00762-x)
Supplement: Supplementary file 2 — Additional file 2. Anova for Regression. [file 12873_2022_762_MOESM2_ESM.docx]

| Additional file 2. Anova for Regression | | | | | | |
| --- | --- | --- | --- | --- | --- | --- |
| Model | | Sum of Squares | df | Mean Square | F | Sig. |
| 1 | Regression | 415.388 | 1 | 415.388 | 3.171 | .077^b^ |
|  | Residual | 25546.470 | 195 | 131.008 |  |  |
|  | Total | 25961.858 | 196 |  |  |  |
| a. Dependent Variable: Job Satisfaction | | | | | | |
| b. Predictors: (Constant), Death Anxiety | | | | | | |
