# Supplementary material for: Investigating the role of predictive death anxiety in the job satisfaction of pre-hospital emergency personnel during the COVID-19 pandemic
Source: BMC Emerg Med. 2022 Dec 6;22:196. doi: 10.1186/s12873-022-00762-x (PMC9727867; doi:10.1186/s12873-022-00762-x)
Supplement: Supplementary file 3 — Additional file 3. A1. Independent Samples Test. A2. Pearson's R. A3. Independent Samples Test. A4. Independent Samples Test. [file 12873_2022_762_MOESM3_ESM.docx]

| Additional file 3A1. Independent Samples Test | | | | | | | | | | |
| --- | --- | --- | --- | --- | --- | --- | --- | --- | --- | --- |
|  | | Levene's Test for Equality of Variances | | t-test for Equality of Means | | | | | | |
|  |  | F | Sig. | t | df | Sig. (2-tailed) | Mean Difference | Std. Error Difference | 95% Confidence Interval of the Difference | |
|  |  |  |  |  |  |  |  |  | Lower | Upper |
| Job Satisfaction | Equal variances assumed | 8.128 | .005 | -.877 | 195 | .381 | -1.97688 | 2.25317 | -6.42058 | 2.46683 |
|  | Equal variances not assumed |  |  | -1.147 | 58.308 | .256 | -1.97688 | 1.72387 | -5.42719 | 1.47343 |
| Death Anxiety | Equal variances assumed | .233 | .630 | -1.334 | 196 | .184 | -.51111 | .38323 | -1.26688 | .24467 |
|  | Equal variances not assumed |  |  | -1.393 | 43.627 | .171 | -.51111 | .36682 | -1.25056 | .22834 |

| Additional file 3A2. Pearson's R | Value | Asymptotic Standard Error^a^ | Approximate T^b^ | Approximate Significance |
| --- | --- | --- | --- | --- |
| Job Satisfaction Age and | .040 | .073 | .558 | .577^c^ |
| Age and Death Anxiety | .016 | .072 | .219 | .827^c^ |

| Additional file 3A3. Independent Samples Test | | | | | | | | | | |
| --- | --- | --- | --- | --- | --- | --- | --- | --- | --- | --- |
|  | | Levene's Test for Equality of Variances | | t-test for Equality of Means | | | | | | |
|  |  | F | Sig. | t | df | Sig. (2-tailed) | Mean Difference | Std. Error Difference | 95% Confidence Interval of the Difference | |
|  |  |  |  |  |  |  |  |  | Lower | Upper |
| Job Satisfaction | Equal variances assumed | 2.814 | .095 | .081 | 192 | .936 | .15261 | 1.89269 | -3.58052 | 3.88574 |
|  | Equal variances not assumed |  |  | .074 | 75.845 | .941 | .15261 | 2.06925 | -3.96878 | 4.27401 |
| Death Anxiety | Equal variances assumed | .004 | .952 | 1.021 | 193 | .308 | .32598 | .31915 | -.30350 | .95546 |
|  | Equal variances not assumed |  |  | 1.010 | 86.047 | .315 | .32598 | .32280 | -.31573 | .96769 |

| Additional file 3A4. Independent Samples Test | | | | | | | | | | |
| --- | --- | --- | --- | --- | --- | --- | --- | --- | --- | --- |
|  | | Levene's Test for Equality of Variances | | t-test for Equality of Means | | | | | | |
|  |  | F | Sig. | t | df | Sig. (2-tailed) | Mean Difference | Std. Error Difference | 95% Confidence Interval of the Difference | |
|  |  |  |  |  |  |  |  |  | Lower | Upper |
| Job Satisfaction | Equal variances assumed | .509 | .476 | 1.498 | 195 | .136 | 2.83246 | 1.89091 | -.89680 | 6.56172 |
|  | Equal variances not assumed |  |  | 1.453 | 78.133 | .150 | 2.83246 | 1.94898 | -1.04756 | 6.71248 |
| Death Anxiety | Equal variances assumed | .007 | .934 | .325 | 196 | .746 | .10459 | .32190 | -.53024 | .73943 |
|  | Equal variances not assumed |  |  | .325 | 84.523 | .746 | .10459 | .32182 | -.53533 | .74451 |
