# Supplementary material for: Investigating the role of predictive death anxiety in the job satisfaction of pre-hospital emergency personnel during the COVID-19 pandemic
Source: BMC Emerg Med. 2022 Dec 6;22:196. doi: 10.1186/s12873-022-00762-x (PMC9727867; doi:10.1186/s12873-022-00762-x)
Supplement: Supplementary file 4 — Additional file 4. Independent Samples Test. [file 12873_2022_762_MOESM4_ESM.docx]

| Additional file 4. Independent Samples Test | | | | | | | | | | |
| --- | --- | --- | --- | --- | --- | --- | --- | --- | --- | --- |
|  | | Levene's Test for Equality of Variances | | t-test for Equality of Means | | | | | | |
|  |  | F | Sig. | t | df | Sig. (2-tailed) | Mean Difference | Std. Error Difference | 95% Confidence Interval of the Difference | |
|  |  |  |  |  |  |  |  |  | Lower | Upper |
| Job Satisfaction | Equal variances assumed | .362 | .548 | 2.599 | 195 | .010 | 5.62435 | 2.16427 | 1.35597 | 9.89274 |
|  | Equal variances not assumed |  |  | 2.733 | 48.201 | .009 | 5.62435 | 2.05831 | 1.48629 | 9.76242 |
| Death Anxiety | Equal variances assumed | .175 | .676 | -.846 | 196 | .399 | -.31313 | .37026 | -1.04333 | .41707 |
|  | Equal variances not assumed |  |  | -.888 | 50.204 | .379 | -.31313 | .35251 | -1.02110 | .39484 |
