# Supplementary material for: Investigating the role of predictive death anxiety in the job satisfaction of pre-hospital emergency personnel during the COVID-19 pandemic
Source: BMC Emerg Med. 2022 Dec 6;22:196. doi: 10.1186/s12873-022-00762-x (PMC9727867; doi:10.1186/s12873-022-00762-x)
Supplement: Supplementary file 5 — Additional file 5. Independent Samples Test. [file 12873_2022_762_MOESM5_ESM.docx]

| Additional file 5. Independent Samples Test | | | | | | | | | | | | | | | | |  |
| --- | --- | --- | --- | --- | --- | --- | --- | --- | --- | --- | --- | --- | --- | --- | --- | --- | --- |
|  | | | Levene's Test for Equality of Variances | | | t-test for Equality of Means | | | | | | | | | | |  |
|  |  |  | F | Sig. | | t | df | | Sig. (2-tailed) | Mean Difference | | Std. Error Difference | 95% Confidence Interval of the Difference | | | |  |
|  |  |  |  |  |  |  |  |  |  |  |  |  | Lower | | Upper | |  |
| Job Satisfaction | Equal variances assumed | | 1.299 | .256 | | 2.570 | 195 | | .011 | 4.38553 | | 1.70664 | 1.01968 | | 7.75139 | |  |
|  | Equal variances not assumed | |  |  | | 2.623 | 141.172 | | .010 | 4.38553 | | 1.67198 | 1.08018 | | 7.69089 | |  |
| Death Anxiety | Equal variances assumed | | .002 | .967 | | -.332 | 196 | | .740 | -.09774 | | .29451 | -.67856 | | .48309 | |  |
|  | Equal variances not assumed | |  |  | | -.333 | 136.930 | | .740 | -.09774 | | .29386 | -.67883 | | .48336 | |  |
| Additional file 6. ANOVA | | | | | | | | | | | | | | | | | |
|  | | | | | Sum of Squares | | | df | | | Mean Square | | | F | | Sig. | |
| Job Satisfaction | | Between Groups | | | 348.552 | | | 3 | | | 116.184 | | | .875 | | .455 | |
|  |  | Within Groups | | | 25613.306 | | | 193 | | | 132.711 | | |  | |  | |
|  |  | Total | | | 25961.858 | | | 196 | | |  | | |  | |  | |
| Death Anxiety | | Between Groups | | | 19.527 | | | 3 | | | 6.509 | | | 1.707 | | .167 | |
|  |  | Within Groups | | | 739.928 | | | 194 | | | 3.814 | | |  | |  | |
|  |  | Total | | | 759.455 | | | 197 | | |  | | |  | |  | |
