# Supplementary material for: Investigating the role of predictive death anxiety in the job satisfaction of pre-hospital emergency personnel during the COVID-19 pandemic
Source: BMC Emerg Med. 2022 Dec 6;22:196. doi: 10.1186/s12873-022-00762-x (PMC9727867; doi:10.1186/s12873-022-00762-x)
Supplement: Supplementary file 6 — Additional file 6. ANOVA. [file 12873_2022_762_MOESM6_ESM.docx]

| Additional file 6. ANOVA | | | | | | |
| --- | --- | --- | --- | --- | --- | --- |
|  | | Sum of Squares | df | Mean Square | F | Sig. |
| Job Satisfaction | Between Groups | 348.552 | 3 | 116.184 | .875 | .455 |
|  | Within Groups | 25613.306 | 193 | 132.711 |  |  |
|  | Total | 25961.858 | 196 |  |  |  |
| Death Anxiety | Between Groups | 19.527 | 3 | 6.509 | 1.707 | .167 |
|  | Within Groups | 739.928 | 194 | 3.814 |  |  |
|  | Total | 759.455 | 197 |  |  |  |
