# Supplementary material for: Investigating the role of predictive death anxiety in the job satisfaction of pre-hospital emergency personnel during the COVID-19 pandemic
Source: BMC Emerg Med. 2022 Dec 6;22:196. doi: 10.1186/s12873-022-00762-x (PMC9727867; doi:10.1186/s12873-022-00762-x)
Supplement: Supplementary file 7 — Additional file 7. ANOVA. [file 12873_2022_762_MOESM7_ESM.docx]

| Additional file 7. ANOVA | | | | | | |
| --- | --- | --- | --- | --- | --- | --- |
|  | | Sum of Squares | df | Mean Square | F | Sig. |
| Job Satisfaction | Between Groups | 2867.249 | 2 | 1433.625 | 12.043 | .000 |
|  | Within Groups | 23094.609 | 194 | 119.044 |  |  |
|  | Total | 25961.858 | 196 |  |  |  |
| Death Anxiety | Between Groups | 2.295 | 2 | 1.147 | .296 | .744 |
|  | Within Groups | 757.160 | 195 | 3.883 |  |  |
|  | Total | 759.455 | 197 |  |  |  |
