# Supplementary material for: Investigating the role of predictive death anxiety in the job satisfaction of pre-hospital emergency personnel during the COVID-19 pandemic
Source: BMC Emerg Med. 2022 Dec 6;22:196. doi: 10.1186/s12873-022-00762-x (PMC9727867; doi:10.1186/s12873-022-00762-x)
Supplement: Supplementary file 8 — Additional file 8. Independent Samples Test. [file 12873_2022_762_MOESM8_ESM.docx]

| Additional file 8. Independent Samples Test | | | | | | | | | | |
| --- | --- | --- | --- | --- | --- | --- | --- | --- | --- | --- |
|  | | Levene's Test for Equality of Variances | | t-test for Equality of Means | | | | | | |
|  |  | F | Sig. | t | df | Sig. (2-tailed) | Mean Difference | Std. Error Difference | 95% Confidence Interval of the Difference | |
|  |  |  |  |  |  |  |  |  | Lower | Upper |
| Job Satisfaction | Equal variances assumed | .096 | .757 | 1.194 | 195 | .234 | 2.07287 | 1.73537 | -1.34965 | 5.49538 |
|  | Equal variances not assumed |  |  | 1.170 | 123.492 | .244 | 2.07287 | 1.77144 | -1.43345 | 5.57918 |
| Death Anxiety | Equal variances assumed | .173 | .678 | .998 | 196 | .319 | .29545 | .29600 | -.28830 | .87921 |
|  | Equal variances not assumed |  |  | 1.005 | 132.601 | .317 | .29545 | .29391 | -.28590 | .87681 |
